# Supplementary material for: Recognition and reprogramming of E3 ubiquitin ligase surfaces by α-helical peptides
Source: Nat Commun. 2023 Nov 1;14:6992. doi: 10.1038/s41467-023-42395-z (PMC10620186; doi:10.1038/s41467-023-42395-z)
Supplement: Supplementary file 1 — Supplementary Information [file 41467_2023_42395_MOESM1_ESM.pdf]

Supplementary Information

Recognition and reprogramming of E3 ubiquitin ligase surfaces by helical peptides

Olena S. Tokareva, Kunhua Li, Tara L. Travaline, Ty M. Thomson, Jean-Marie Swiecicki, Mahmoud Moussa, Jessica D. Ramirez, Sean Litchman, Gregory L. Verdine, and John H. McGee

Supplementary Figures:

Supplementary Figure 1. Helicon binding and biochemical activity against WWP members of the HECT E3 family

Supplementary Figure 2. Helicon binding to CRL family E3 ligases

Supplementary Figure 3. Identification of Helicon residues responsible for binding to MDM4 and CHIP

Supplementary Figure 4. Biochemical characterization of CHIP and MDM2 trimerizer Helicons

Supplementary Figure 5 – Determination of the binding kinetics and degree of complex formation of trimerizer Helicons that induce the interaction of MDM2 and β-catenin, CHIP and PPIA, and CHIP and TEAD4 by SPR (ABA mode).

Supplementary Figure 6. Structural characterization of Helicon:MDM2:β-catenin ternary complexes

Supplementary Figure 7 – Electron density maps of Helicons (*2mFo-dFc*, 1.0 σ) bound to targets studied in this work.

Supplementary Figure 8 – Raw biochemical data supporting Figures 4a and 4c, and Supplementary Figures 2b, 4a-d.

Supplementary Tables:

Supplementary Table 1 – List of all peptides in this study

Supplementary Table 2 – Primer sequences used to build trimerizer libraries

Supplementary Table 3 – Macromolecular interfaces driven by trimerizer Helicons

Supplementary Figures:

a

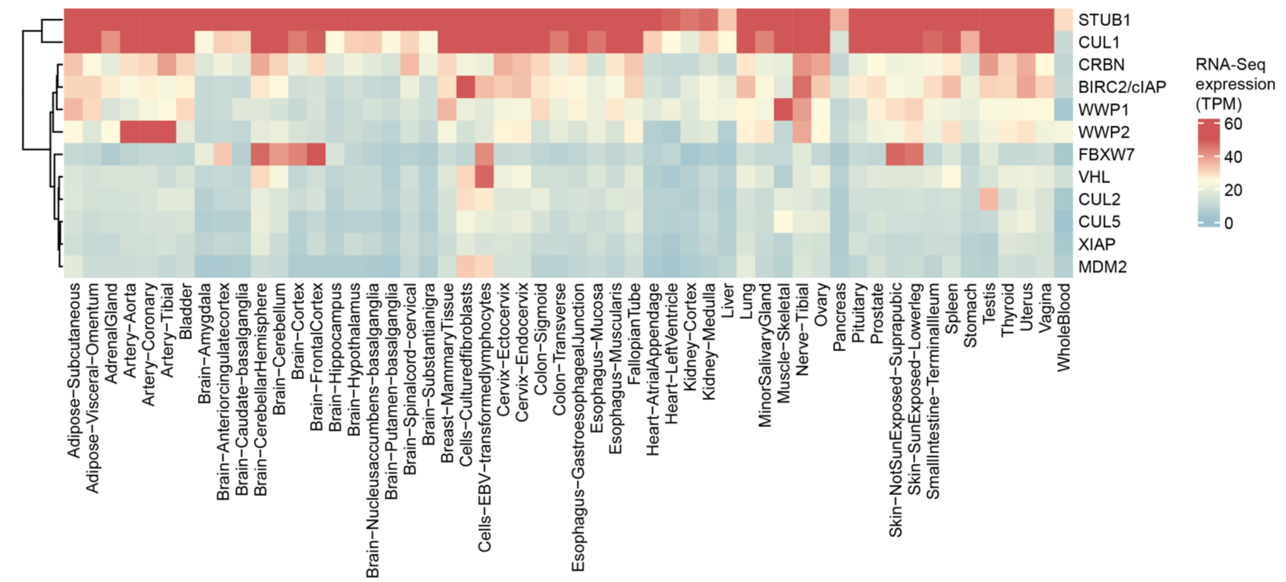

b

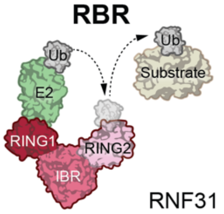

c

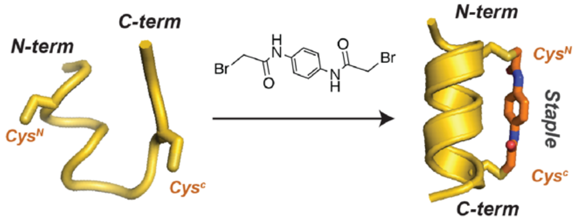

d

Parent Cluster	Peptide ID	Sequence	K_D (SPR, μM) WWP1$^{WW-HECT}$	WWP1^{HECT}	WWP2^{HECT}
Group-1 pan-WWP (Cluster C71)	Helicon H302	DPASMRCHRAAHICSVL	7.2	9.8	7.7
	Helicon H303	DPAMYHCEQAANVCRVL	15.7	8.5	7.8
	Helicon H301	DPADRRCIQAARVCAVL	4.3	4.5	3.5
Group-2 pan-HECT (Cluster C73)	Helicon H308	DPANWECRYAAFNCFIQ	>20	8.8	1.3
	Helcion H309	DPAAWDCLYAAWDCYIN	>20	>20	8.0
	Helicon H304#	DPAQMYCREAAFYCFMH	>20	12.1	1.2
Group-3 WWP2-HECT (Cluster C72)	Helicon H305	DPATIMCRAAADTCTFF	>20	>20	0.69
	Helicon H306	DPAYHHCLAAADLCSFF	>20	>20	0.39
	Helicon H307	DPADQWCLAAADVCHFF	>20	>20	0.78
Group-4 WWP1-WW (Cluster C74)	Helicon H310	DPALQACTHAANLCHHF	4.5	>20	>20
	Helicon H311	DPALMQCAHAAVLCHQF	17.4	>20	>20
	Helcion H312	DPATQRCSMAAYMCHTT	20.0	>20	>20

e

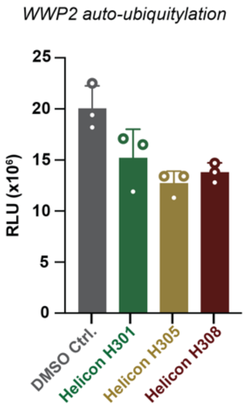

f

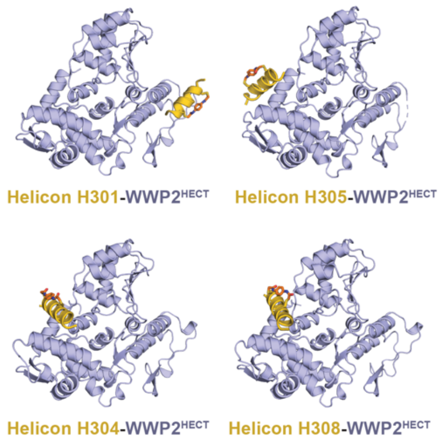

g

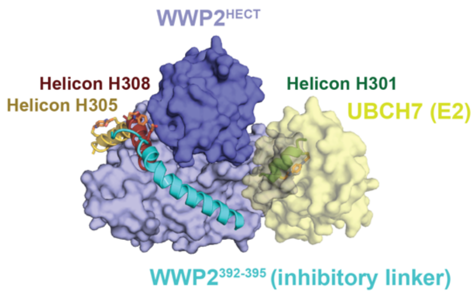

Supplementary Figure 1. Helicon binding and biochemical activity against WWP members of the HECT E3 family

a Heatmap of E3 ligase gene expression across adult normal tissues using RNA-Seq data from the GTEx Portal (gtexportal.org). Colors represent the median expression in transcripts per million (TPM) in indicated tissue. **b** A prototypical RBR E3 ligase highlighting the two RING domains of this family. Helicons directly binding RBR E3 RNF31 were described previously[1]. **c** Cysteine stapling (orange) with a bifunctional bromoacetamide cross-linker stabilizes the α-helical conformation of Helicons[1]. **d** SPR validation of hits derived from screening against three HECT-containing proteins defines four groups of Helicons based on their binding to both inactive and active forms of both HECT domains (Group-1), to both isolated WWP1 and WWP2 HECT domains (Group-2), to the WWP2 HECT domain ($WWP2^{HECT}$) only (Group-3), or to the WW domain only (Group-4) (n=2). [#]The Group-2 logo shown (red) represents Cluster C73 (C73), but the C71 logo (including from H304) is similar. **e** Helicons from Groups-1 through -3 could partially inhibit the in vitro auto-ubiquitylation activity of $WWP2^{HECT}$. n = 3; data are presented as mean ± SD of biologically independent samples. **f** X-ray co-crystal structures of Helicons from Groups-1 through -3 with $WWP2^{HECT}$ as in Fig. 2c, and including the H304-bound WWP2 (PDB: 8EI7). **g** The structural overlay shown in Fig. 2c, with the addition of the WWP2 helix residues 382-395 that form the inhibitory linker domain of WWP2 (aqua, PDB: 5TJQ[2]) and the E2 UBCH7 (yellow, PDB: 5HPT[3]) at the Helicon H301-binding interface with $WWP2^{HECT}$. Electron density maps of these Helicons are shown in Supplementary Fig. 7. Source data are provided as a Source Data file.

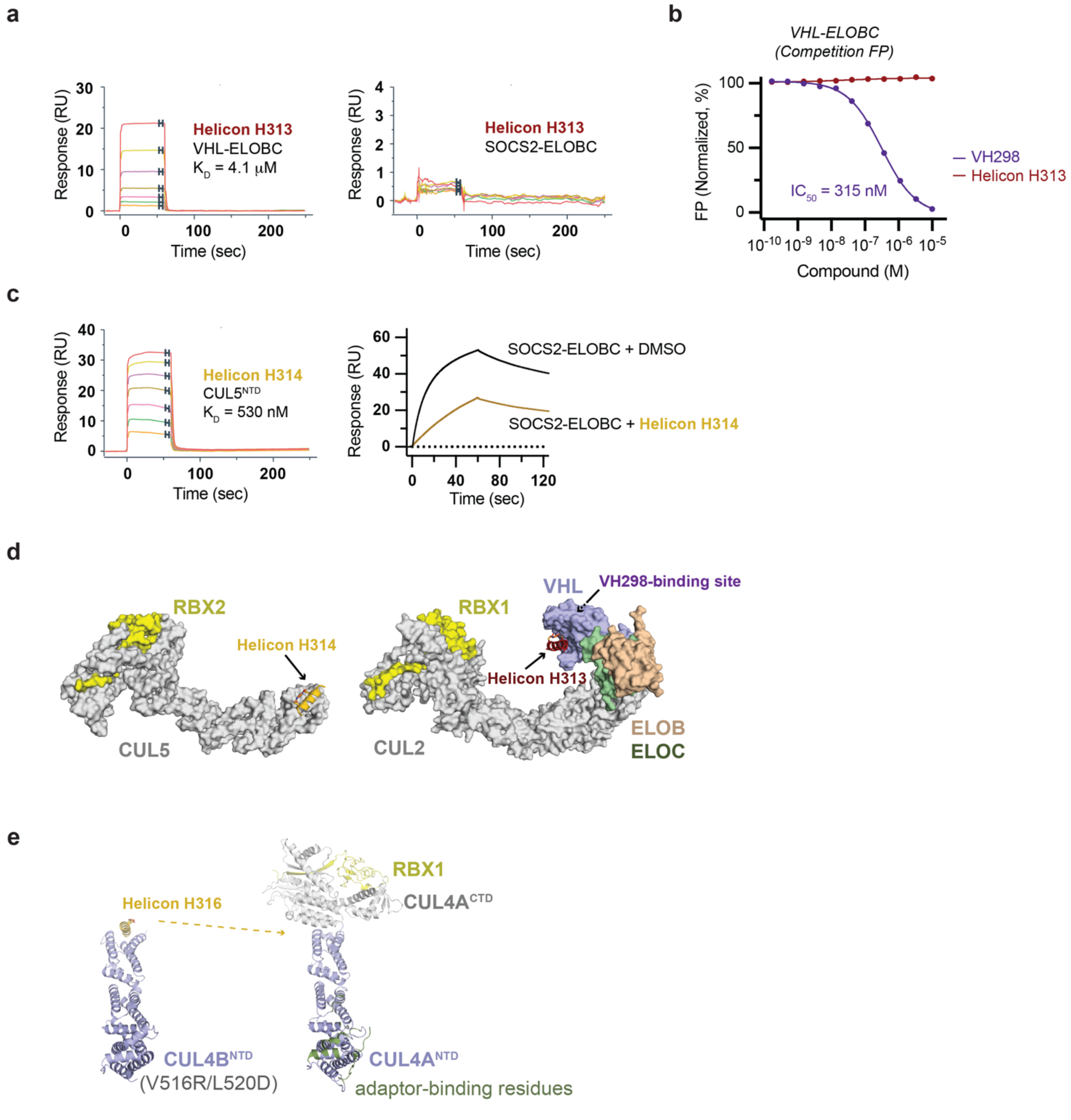

Supplementary Figure 2. Helicon binding to CRL family E3 ligases

a SPR analysis of Helicon H313 revealing its specificity for VHL-ELOBC (left) over SOCS2-ELOBC (right). n = 1. **b** A competition FP assay was performed using VHL-ELOBC and VHL-binding fluorescent probe HXC78 (ref. [4]), revealing that Helicon H313 does not compete with HXC78 for binding VHL-ELOBC, while VH298 – a molecule related to HXC78 (ref. [5]) – potently competes (n = 2; data are presented as mean of technical replicates). Data were normalized as described for Fig. 4c. VH298 binds VHL with a K_D of 90 nM[5]. Competition FP data that have not been normalized are graphed in Supplementary Fig. 8a. **c** SPR analysis of H314 binding to CUL5 (left) and a competition SPR (ABA injection mode) assay (right) reveal that H314 binds to CUL5 to disrupt its interaction with SOCS2-

ELOBC. (n = 1). **d** H314 overlaid on the full-length CUL5 bound to RING-box protein 2 (CUL5-RBX2, PDB 6V9I[6]) using PyMol (left). The surface of CUL5 and RBX2 are shown in grey and yellow, respectively. Helicon H313 was overlaid on the CUL2-RBX1-ELOBC-VHL E3 ligase complex (PDB 5N4W[7]) using PyMol, with CUL2 and RBX1 shown in grey and yellow, respectively. The small molecule VHL ligand, VH298, binds on the opposite side from Helicon H313 (PDB 5LLI[5]) (right). **e** Helicon H316 was crystallized with the CUL4B N-terminal domain ($CUL4B^{NTD}$) that also harbors V516R/L520D point mutations near the truncation site to assist with protein expression – as reported for CUL1 (ref. [8]) – and the co-structure was solved at 2.89Å resolution (PDB: 8EI1) (left). The corresponding Helicon H316-binding site on CUL4A, which shares an 81% sequence identity with CUL4B, is located at the interface between $CUL4A^{NTD}$ and the C-terminal domain ($CUL4A^{CTD}$), indicating a non-physiological Helicon interaction (right). The overlaid $CUL4A^{NTD}$ structure is from PDB 2HYE[9]. Electron density maps of these E3-bound Helicons are shown in Supplementary Fig. 7. Source data are provided as a Source Data file.

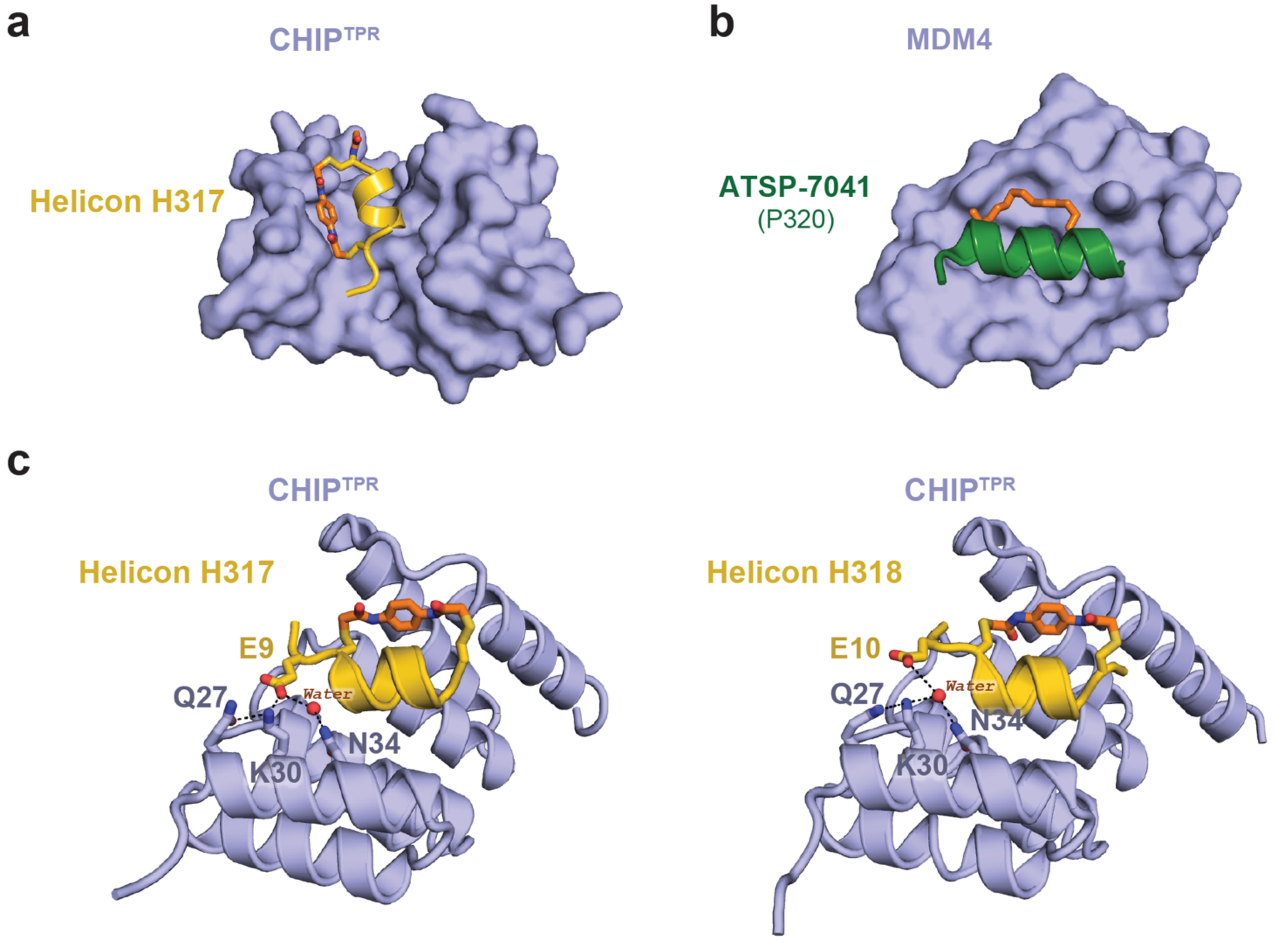

Supplementary Figure 3. Identification of Helicon residues responsible for binding to MDM4 and CHIP

a An additional H317-CHIP co-structure view. Helicon H317 was crystallized with the TPR domain of CHIP (residues 23-154, $CHIP^{TPR}$), as for Fig. 3c, and the co-structure was solved at 2.06 Å resolution (PDB: 8EHZ). **b** Published structure of ATSP-7041 (referred to here as P320) with MDM4 (PDB 4N5T[10]). **c** Comparison of the H317-$CHIP^{TPR}$ (residues 21-154) structure and the 1.47 Å resolution structure of H318. The two Helicons are from the same CHIP-binding cluster, including a shared E10 residue. E9 of H317 directly interacts with CHIP whereas E10 of H318 does not, suggesting regional plasticity of the interaction between Helicon and protein. Electron density maps of these Helicons are shown in Supplementary Fig. 7.

CHIP TEAD trimerizers

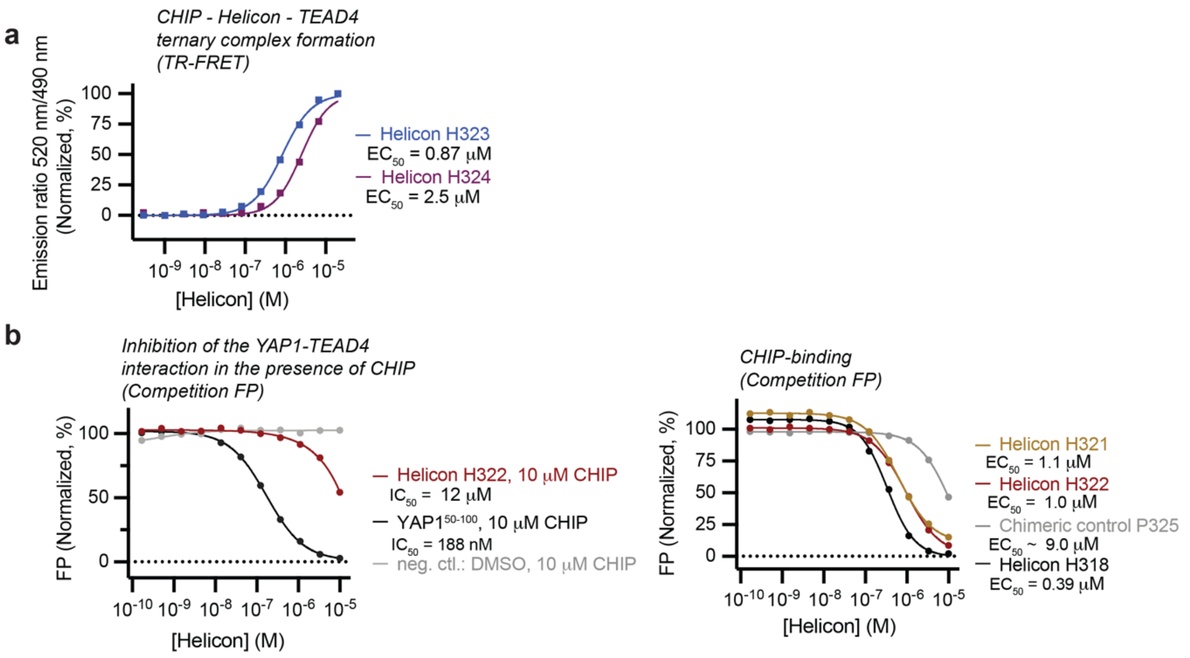

MDM2 β-catenin trimerizers

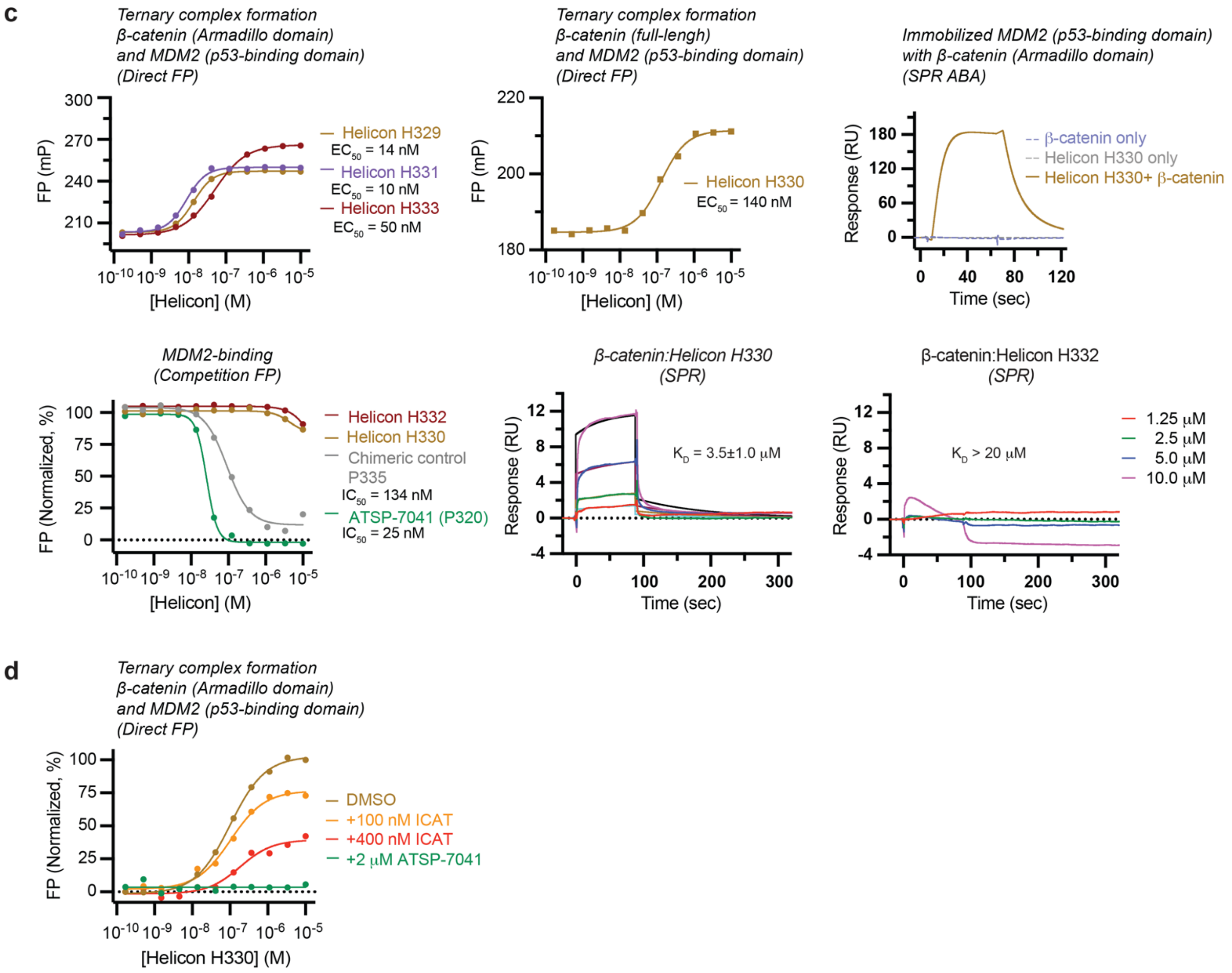

Supplementary Figure 4. Biochemical characterization of CHIP and MDM2 trimerizer Helicons

a TR-FRET was performed as in Fig. 4a to assess H323- and H324-mediated CHIP:TEAD4 ternary complex formation. n = 2; data are presented as mean of technical replicates. Data were normalized as described in Fig. 4a and shown not normalized in Supplementary Fig. 8c. **b** TEAD4 competition FP was performed as in Fig. 4c to demonstrate inhibition of the TEAD4-YAP1 interaction by H322 in the presence of CHIP with additional positive and negative controls (left). Competition FP to evaluate CHIP interactions with H321 and a C-terminally truncated version of H321 (H322) (right). n = 2; data are presented as the mean of technical replicates. Data were normalized as described in Fig. 4c and shown not normalized in Supplementary Figs. 8f and 8g. **c** Direct FP was performed as in Fig. 5b to assess ternary complex formation mediated by MDM2:β-catenin trimerizers H329, H333, and an N-terminally truncated version of H329 (H331) using the β-catenin Armadillo domain (top left). Direct FP assays reveal ternary complex formation mediated by H330 (top middle). n = 2; data are presented as the mean of technical replicates. Data were normalized as described in Fig. 4a. SPR (ABA injection mode) assay using immobilized MDM2. n = 1 (top right). Additional characterization of Helicons via MDM2-competition FP. Control peptides ATSP-7041 and P335 interact with MDM2. H330 and H332 only interact with the MDM2 p53-binding domain with a low affinity (IC_{50} > 10 μM, bottom left). n = 2; data are presented as the mean of technical replicates. Data were normalized as described in Fig. 4c. SPR assays performed as in Fig. 5c to assess the interaction of H330 and H332 with immobilized β-catenin. n = 1 (bottom middle and right). Data that have not been normalized are graphed in Supplementary Fig. 8h. **d** The direct FP assay to assess ternary complex formation by H330. n = 2; data are presented as the mean of technical replicates. Data were normalized as described in Fig. 4a and shown not normalized in Supplementary Fig. 8i. Source data are provided as a Source Data file.

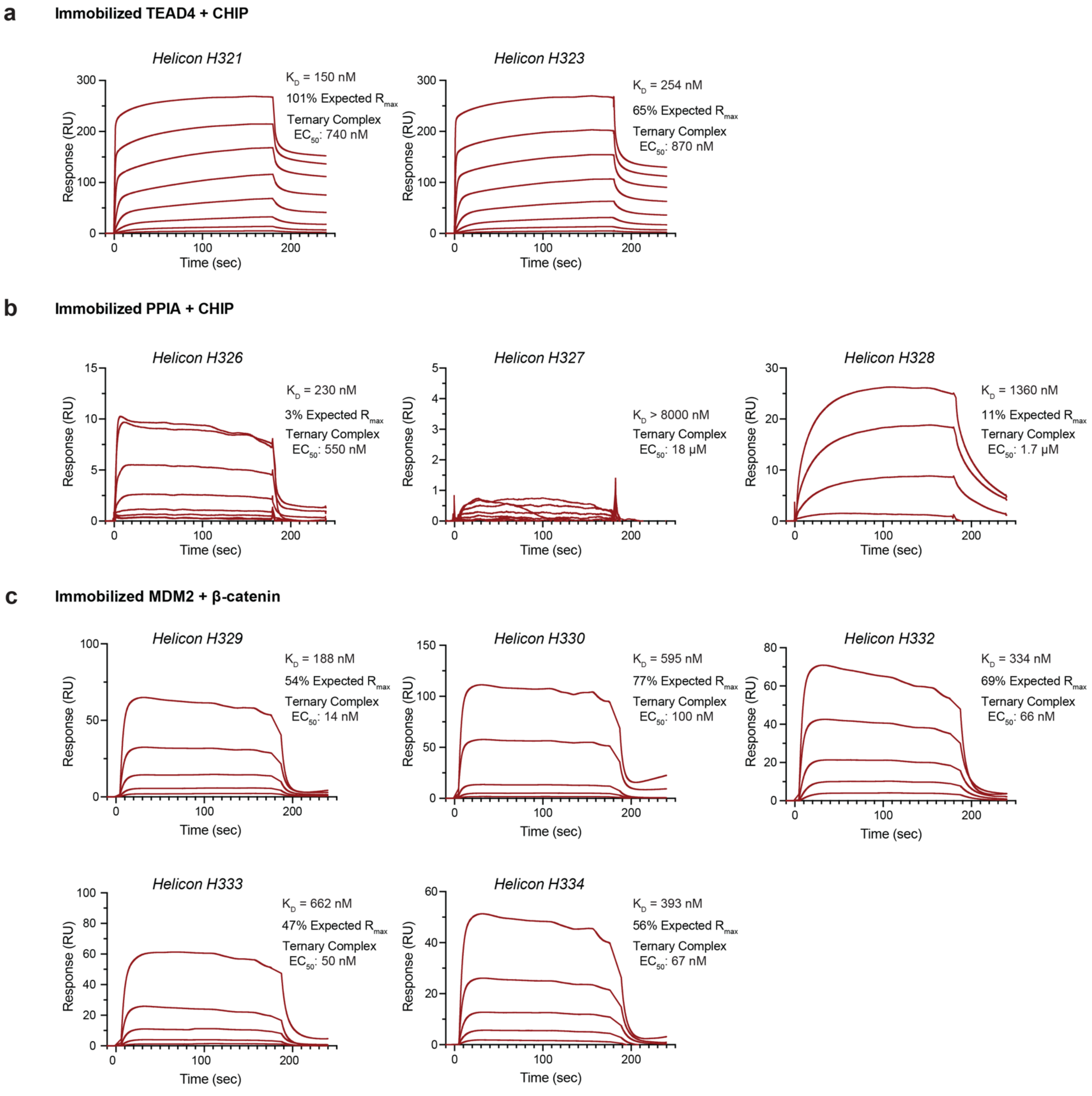

Supplementary Figure 5. Determination of the binding kinetics and degree of complex formation of trimerizer Helicons that induce the interaction of MDM2 and β-catenin, CHIP and PPIA, and CHIP and TEAD4 by SPR (ABA mode).

a SPR was performed by immobilizing TEAD4 onto a sensor surface, then injecting several concentrations of CHIP in the presence of 10 μM Helicon H321 (left) or Helicon H323 (right). K_D values with which Helicons induced ternary complexes are shown. The molecular weights of each component of the ternary complex were used to calculate the expected response in RU, which was then divided by

the experimental response (R_{max}) to determine the amount of ternary complex formed compared to the theoretical maximum amount possible based on this response. **b** SPR experiments and calculations for the PPIA/CHIP complex were performed as for panel A, but with immobilized PPIA and injections of CHIP at several concentrations in the presence of 10 µM Helicon H326 (left), Helicon H327 (center), or Helicon H328 (right). **c** SPR experiments and calculations for the MDM2:β-catenin complex were performed as for panel A, but with immobilized MDM2 and injections of β-catenin at several concentrations in the presence of 10 µM Helicon H329 (top left), Helicon H330 (top center), Helicon H332 (top right), Helicon H333 (bottom left) or Helicon H334 (bottom right). Source data are provided as a Source Data file.

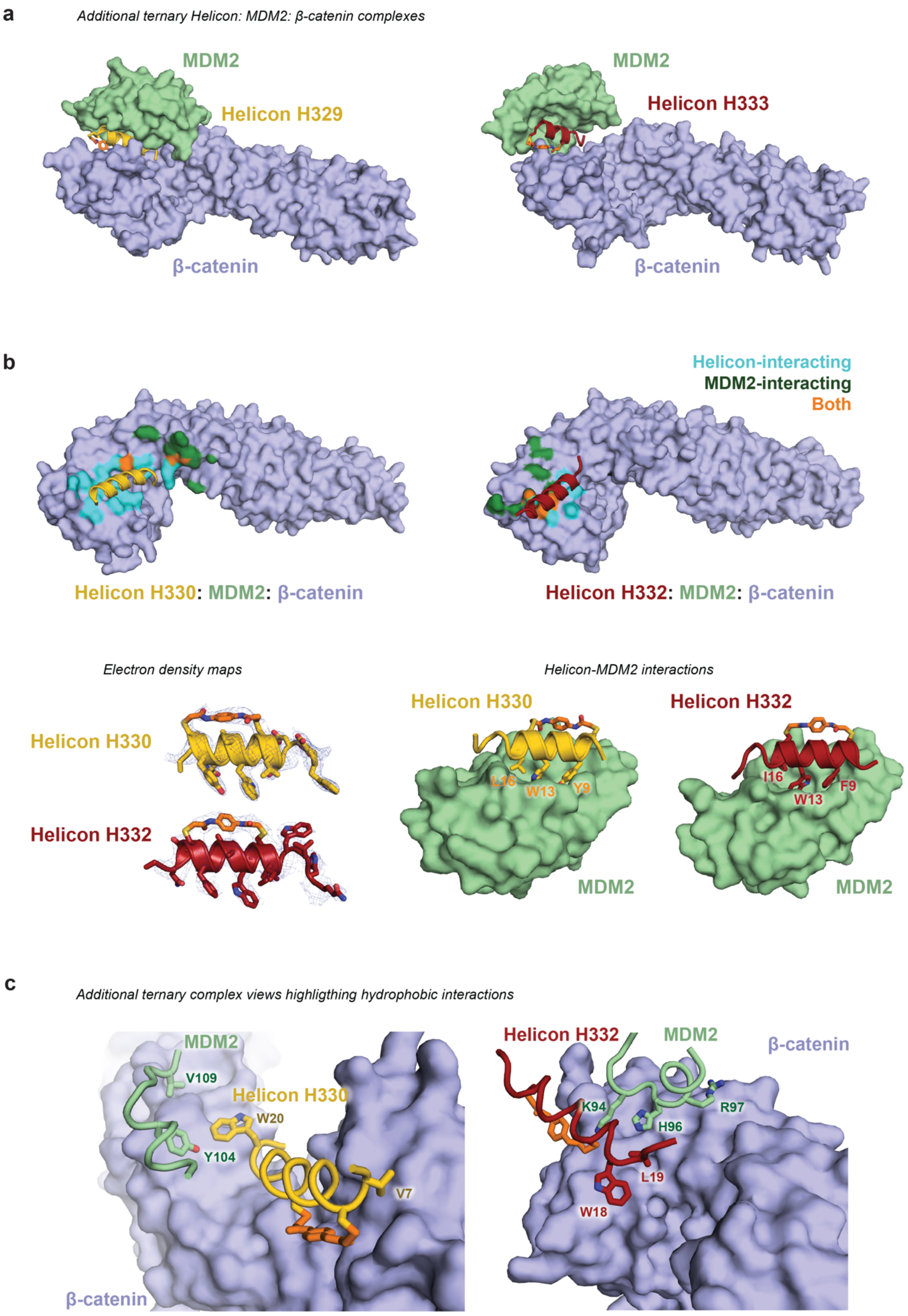
a
Additional ternary Helicon: MDM2: β-catenin complexes
MDM2
Helicon H329
β-catenin
MDM2
Helicon H333
β-catenin
b
Helicon-interacting
MDM2-interacting
Both
Helicon H330: MDM2: β-catenin
Helicon H332: MDM2: β-catenin
Electron density maps
Helicon H330
Helicon H332
Helicon-MDM2 interactions
Helicon H330
L16
W13
Y9
MDM2
Helicon H332
I16
W13
F9
MDM2
c
Additional ternary complex views highligthing hydrophobic interactions
MDM2
V109
Helicon H330
W20
Y104
V7
β-catenin
Helicon H332
MDM2
β-catenin
K94
H96
R97
L19
W18

Supplementary Figure 6. Structural characterization of Helicon:MDM2:β-catenin ternary complexes

a X-ray co-crystal structures of the complexes between β-catenin and MDM2 mediated by two additional Helicons, H329 from C91 and H333 from C92 (PDB: 8EIB and 8EIA, respectively). **b** Both Helicon and MDM2 surfaces contribute to binding to β-catenin and thus ternary complex formation. The Helicon- and MDM2-adjacent surfaces (within 4.5 Å distance) of β-catenin are highlighted in teal and green, respectively, and orange for surfaces adjacent to both Helicon and MDM2. Electron density maps of Helicons (2*mFo*-*dFc*, 1.0 σ) and isolated MDM2-Helicon interactions. **c** Additional views of ternary complexes induced by Helicons 330 and 332. Electron density maps of these Helicons are shown in Supplementary Fig. 7.

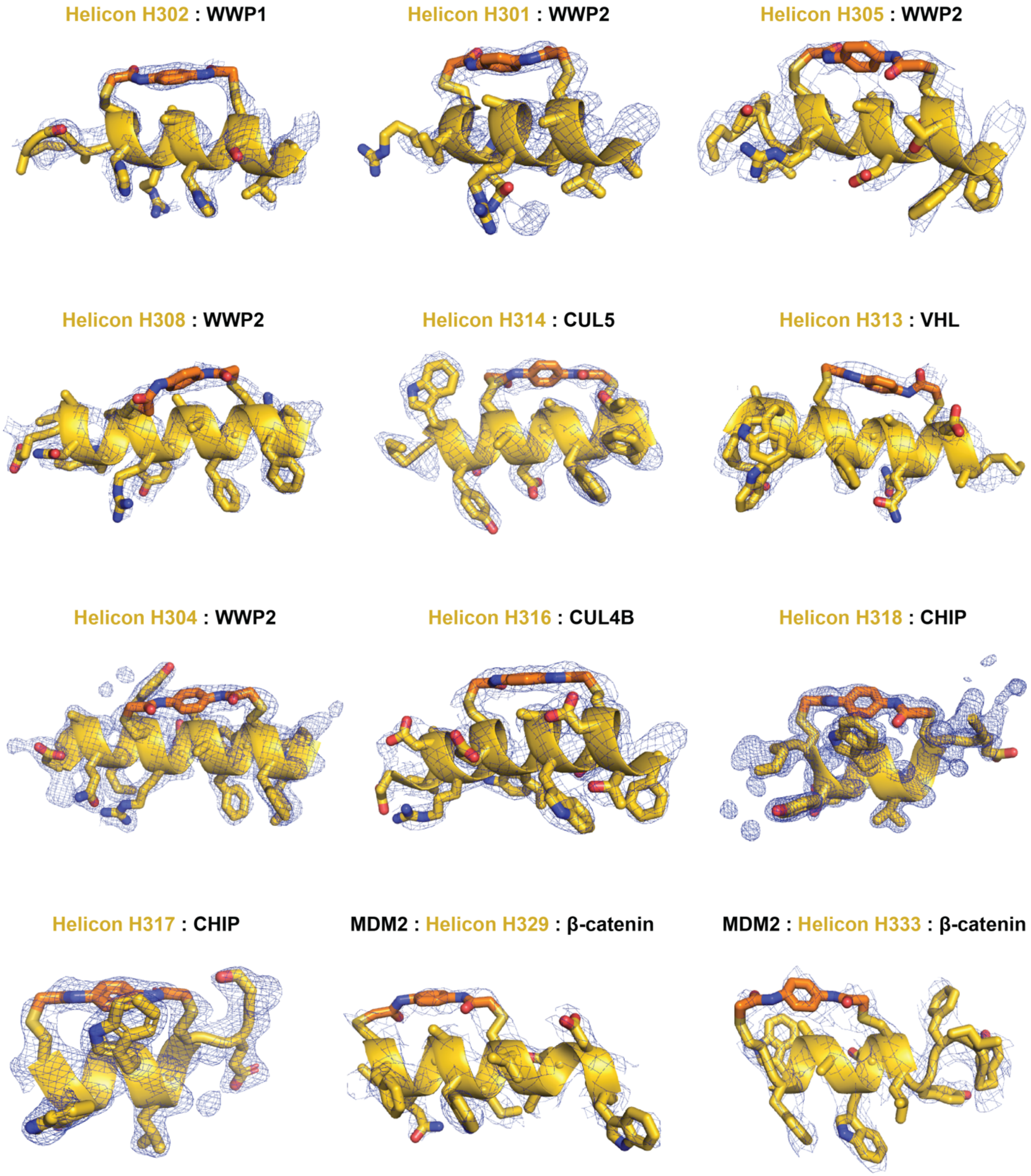

Supplementary Figure 7. Electron density maps of Helicons (*2mFo-dFc*, 1.0 σ) bound to targets studied in this work. PDB codes corresponding to each target-bound Helicon structures are 8EI4 (WWP1-bound Helicon H302), 8EI5 (WWP2-bound H301), 8EI6 (WWP2-bound H305), 8EI8 (WWP2-bound H308), 8EI2 (CUL5-bound H314), 8EI3 (VHL-bound H313), 8EI7 (WWP2-bound H304), 8EI1 (CUL4B-bound H316), 8EI0 (CHIP-bound H318), 8EHZ (CHIP-bound H317), 8EIB (H329 bound to MDM2 and β-catenin), and 8EIA (H333 bound to MDM2 and β-catenin). Electron density maps of additional Helicons (H329 and H333) bound to MDM2 and β-catenin are shown in Extended Data Figure 6b, corresponding to PDB codes 8EIB and 8EIA, respectively.

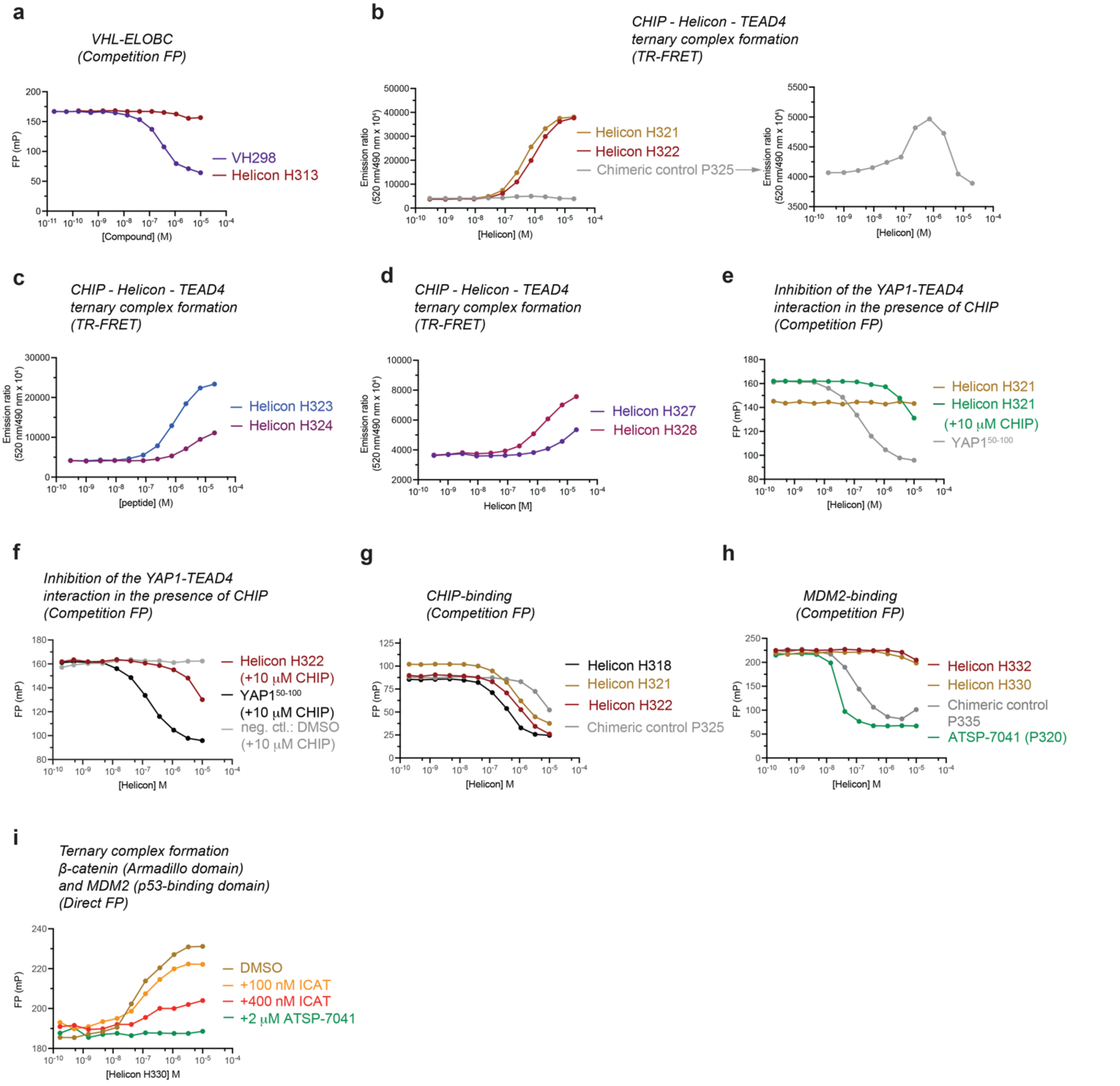

Supplementary Figure 8. Raw biochemical data for ternary complexes supporting Figures 4a and 4c, and Supplementary Figures 2b and 4a-d. Competition FP experiments were performed as in Figure 4c and Supplementary Figures 2b, 4b, and 4c. TR-FRET experiments were performed as in Figure 4a and Supplementary Figure 4a. The Direct FP experiment was performed as in Supplementary Figure 4d. **a** Raw data (not normalized, in absolute units) corresponding to the Competition FP experiment shown in Supplementary Figure 2b. **b** Raw data from the same experiment corresponding to the normalized TR-FRET data shown in Figure 4a. The data are shown in two different scales to better visualize the magnitude of the responses with Helicons H321 and H322. **c** Raw data corresponding to the TR-FRET experiment shown in Supplementary Figure 4a. **d** Raw data corresponding to the TR-

FRET experiment shown in Figure 4a. **e** Raw data corresponding to the Competition FP experiment shown in Figure 4c. (**f**, **g**) Raw data corresponding to the Competition FP experiments shown in Supplementary Figure 4b. **h** Raw data corresponding to the Competition FP experiment shown in Supplementary Figure 4c. **i** Raw data corresponding to the TR-FRET experiment shown in Supplementary Figure 4d. Source data are provided as a Source Data file.

Supplementary Table 1 – List of all peptides in this study

Target	Helicon Name	Cluster Name	Sequence	Modification	Expected Mass	Observed Mass
WWP1/WWP2	H301	C71	Ac-DPADRRCIQAARVCAVL-NH2	Cys-stapled	2086.395	2086.4
WWP1/WWP2	H302	C71	Ac-DPASMRCHRAAHICSVL-NH2	Cys-stapled	2096.413	2094.9
WWP1/WWP2	H303	C71	Ac-DPAMYHCEQAANVCRVL-NH2	Cys-stapled	2149.426	2148.0
WWP1/WWP2	H304	C71	Ac-DPAQMYCREAAFYCFMH-NH2	Cys-stapled	2310.911	2312.2
WWP1/WWP2	H305	C72	Ac-DPATIMCRAAADTCTFF-NH2	Cys-stapled	2063.331	2062.6
WWP1/WWP2	H306	C72	Ac-DPAYHHCLAAADLCSFF-NH2	Cys-stapled	2110.324	2109.8
WWP1/WWP2	H307	C72	Ac-DPADQWCLAAADVCHFF-NH2	Cys-stapled	2138.334	2138.2
WWP1/WWP2	H308	C73	Ac-DPANWECRYAAFNCFIQ-NH2	Cys-stapled	2277.489	2277.5
WWP1/WWP2	H309	C73	Ac-DPAAWDCLYAAWDCYIN-NH2	Cys-stapled	2219.404	2218.6
WWP1/WWP2	H310	C74	Ac-DPALQACTHAANLCHHF-NH2	Cys-stapled	2078.288	2077.2
WWP1/WWP2	H311	C74	Ac-DPALMQCAHAAVLCHQF-NH2	Cys-stapled	2084.398	2084.1
WWP1/WWP2	H312	C74	Ac-DPATQRCSMAAYMCHTT-NH2	Cys-stapled	2116.375	2116.2
VHL	H313	C75	Ac-DPAWWNCFSAAQQCDAM-NH2	Cys-stapled	2173.360	2172.6
CUL5	H314	C76	Ac-DPAWYDCADAAWICTFQ-NH2	Cys-stapled	2205.377	2206.8
CUL4B	H316	C77	Ac-DPADRWCELAAWTCDTF-NH2	Cys-stapled	2229.400	2228.2
CHIP	H317	C80	Ac-CWEAWLLCET-NH2	Cys-stapled	1482.677	1482.7
CHIP	H318	C80	Ac-PCYEAWVLCEY-NH2	Cys-stapled	1604.798	1604.8
MDM2	H319	C81	Ac-DPANHACFQAAWDCQFF-NH2	Cys-stapled	2200.364	2200.4
MDM2	P320		Ac-LTF-R8-EYWAQ-Cba-S5-SAA-NH2	R8-S5 stapled	1745.020	1745.3
CHIP-TEAD4	H321	C87	Ac-PVPFFWECQYAAATCQTPRIK-NH2	Cys-stapled	2686.066	2685.5
CHIP-TEAD4	H322	C87	Ac-PVPFFWECQYAAATCQ-NH2	Cys-stapled	2090.333	2089.4
CHIP-TEAD4	H323	C87	Ac-PTPFFWECQFAAATCTAPRVQ-NH2	Cys-stapled	2600.919	2600.8
CHIP-TEAD4	H324	C87	Ac-PVPFFWDCQFAAATCDAPQRR-NH2	Cys-stapled	2655.957	2655.9
CHIP-TEAD4	P325		Ac-LWWPDGSGSGGSPGQVPMRKRQLPASFWEEPR-NH2	unstapled	3638.026	3637.5
CHIP-PPIA	H326	C88	Ac-PAQDDWSCVEAAYLCENYVRV-NH2	Cys-stapled	2660.883	2661.0
CHIP-PPIA	H327	C89	Ac-PILQGMACGPAATICWIDGII-NH2	Cys-stapled	2372.819	2372.8
CHIP-PPIA	H328	C89	Ac-PDMLAPMCGPAASICWIDGVI-NH2	Cys-stapled	2389.827	2389.8
MDM2-β-catenin	H329	C91	Ac-PMEQQAICFQAAWMCLADDWT-NH2	Cys-stapled	2688.037	2688.4
MDM2-β-catenin	H330	C91	Ac-PWKYEQVCYQAAWQCLSDDWD-NH2	Cys-stapled	2864.078	2863.7
MDM2-β-catenin	H331	C91	Ac-QAICFQAAWMCLADDWT-NH2	Cys-stapled	2202.484	2201.6
MDM2-β-catenin	H332	C92	Ac-PISAANDCFKAAWQCIIWLHQ-NH2	Cys-stapled	2645.017	2645.1
MDM2-β-catenin	H333	C92	Ac-PSENARDCFWAAWDCLYFIYQ-NH2	Cys-stapled	2828.091	2827.6

MDM2-β-catenin	H334	C93	Ac-PNLTGADCFPAAWQCLQFLWD-NH2	Cys-stapled	2625.925	2625.8
MDM2-β-catenin	P335		Ac-PD-cyclopentylalanine-CDDAAFNC-3Thi-benzothienylalanine-QGSGS-bAla-LTFEHYWAQLTS-NH2	Cys-stapled	3760.075	3758.7

Supplementary Table 2 – Primer sequences used to build trimerizer libraries

Name	Sequence	Presenter
PR01	CATGCCCGGGTACCTTTCTATTCTCACTCTGCGCCGXXXXXXTGTXXgcagcaASCATTT<br>GTATGGTTGATXXXggtggttctggcgcaggtcgtggttc	CHIP
PR02	CATGCCCGGGTACCTTTCTATTCTCACTCTGCGCCGXXXXXXTGTXXgcagcaASCATTT<br>GTTGGATTGATXXXggtggttctggcgcaggtcgtggttc	CHIP
PR03	CATGCCCGGGTACCTTTCTATTCTCACTCTGCGCCGXXXXXXTGTXXgcagcaGAGRTTT<br>GTTGGMTTTATXXXggtggttctggcgcaggtcgtggttc	CHIP
PR04	CATGCCCGGGTACCTTTCTATTCTCACTCTGCGCCGXXXXXXTGTXCATgcagcaGATRT<br>KTGTTGGMTTTWTXXXggtggttctggcgcaggtcgtggttc	CHIP
PR05	CATGCCCGGGTACCTTTCTATTCTCACTCTGCGCCGXXXTWTGAKXTGTXXgcagcaATT<br>AKGTGTMTTGTTXXXXggtggttctggcgcaggtcgtggttc	CHIP
PR06	CATGCCCGGGTACCTTTCTATTCTCACTCTGCGCCGXXXTTTTGGXTGTCAGTWTgca<br>gcaGCGAYGTGTXXXXXXggtggttctggcgcaggtcgtggttc	CHIP
PR07	CATGCCCGGGTACCTTTCTATTCTCACTCTGCGCCGXXXXTATXTGTATGGAGgca<br>gcaSTGCTGTGTAYGXXXXXggtggttctggcgcaggtcgtggttc	CHIP
PR08	CATGCCCGGGTACCTTTCTATTCTCACTCTGCGCCGXXXXTGGAGCTGTGYGGAGgca<br>gcaTWTMTRTGTGAKXYWTXXXggtggttctggcgcaggtcgtggttc	CHIP
PR09	CATGCCCGGGTACCTTTCTATTCTCACTCTGCGCCGXXXASTATTTGGTGTRTTGATgca<br>gcaXXTGTXXXXXXggtggttctggcgcaggtcgtggttc	CHIP
PR10	CATGCCCGGGTACCTTTCTATTCTCACTCTGCGCCGXXXXXMTGTGTATTTGGgca<br>gcaGATGATTGTXXXXXXggtggttctggcgcaggtcgtggttc	CHIP
PR11	CATGCCCGGGTACCTTTCTATTCTCACTCTGCGCCGXXXASCGATXTGTGTGTATgca<br>gcaTATTTCTGTGAKXXXXXggtggttctggcgcaggtcgtggttc	CHIP
PR12	CATGCCCGGGTACCTTTCTATTCTCACTCTGCGCCGXXXXXXTGTXXGCGAGCMTTTGG<br>TGTRTTGATXXXXggtggttctggcgcaggtcgtggttc	CHIP
PR13	CATGCCCGGGTACCTTTCTATTCTCACTCTGCGCCGXXXXXXTGTTGGGAKGCGTGGV<br>TTXTGTGAAXXXXXggtggttctggcgcaggtcgtggttc	CHIP
PR14	CATGCCCGGGTACCTTTCTATTCTCACTCTGCGCCGXXXXXXTGTTWTXGCAGCATTT<br>XTGTVTTXGATXXXggtggttctggcgcaggtcgtggttc	MDM2
PR15	CATGCCCGGGTACCTTTCTATTCTCACTCTGCGCCGXXXXXGATTGTTTTXGCAGCATG<br>GXTGTWTKXXXXXggtggttctggcgcaggtcgtggttc	MDM2
PR16	CATGCCCGGGTACCTTTCTATTCTCACTCTGCGCCGXXXSAAXXTGTTWTCAAGCAGCAT<br>GGXTGTVTTXGATXXXggtggttctggcgcaggtcgtggttc	MDM2
PR17	CATGCCCGGGTACCTTTCTATTCTCACTCTGCGCCGXXXXXXTGTTTTTGGGCAGCATGG<br>XTGTXXXXXXggtggttctggcgcaggtcgtggttc	MDM2
PR18	CATGCCCGGGTACCTTTCTATTCTCACTCTGCGCCGXXXXXXTGTTTTCAGGCAGCATGG<br>GATTGTCAGTWTTGGXXXggtggttctggcgcaggtcgtggttc	MDM2
PR19	CATGCCCGGGTACCTTTCTATTCTCACTCTGCGCCGXXXXXXTGTTTTCAGGCAGCATGG<br>GATTGTCAGTWTTTTXXXggtggttctggcgcaggtcgtggttc	MDM2
PR20	CATGCCCGGGTACCTTTCTATTCTCACTCTGCGCCGXXXXXXTGTXGATGCAGCATTTXT<br>GTXTWTTATXXXggtggttctggcgcaggtcgtggttc	MDM2
PR21	CATGCCCGGGTACCTTTCTATTCTCACTCTGCGCCGXXXXTGGGAATGTXGAAGCAGCA<br>WTKXTGTXTWTTWTXXXggtggttctggcgcaggtcgtggttc	MDM2
PR22	CATGCCCGGGTACCTTTCTATTCTCACTCTGCGCCGXXXXTGGXTGTTGGGAKGCAGCA<br>XXTGTXGAKXXXXggtggttctggcgcaggtcgtggttc	MDM2
PR23	CATGCCCGGGTACCTTTCTATTCTCACTCTGCGCCGXXXXXXTGTXXGCAGCATTTXTGT<br>XTGGXXXXggtggttctggcgcaggtcgtggttc	MDM2
PR24	CATGCCCGGGTACCTTTCTATTCTCACTCTGCGCCGXXXXXXTGTTWTXGCAGCATTTXT<br>GTVTTXGATXXXggtggttctggcgcaggtcgtggttc	MDM2
PR25	CATGCCCGGGTACCTTTCTATTCTCACTCTGCGCCGXXXXTGGXTGTACCASTGCAGCARTTXTGTXTWTXXXXgg<br>tggttctggcgcaggtcgtggttc	MDM2

Mixed-base code	Mixed Bases
R	A, G
Y	C, T
M	A, C
K	G, T

S	G, C
W	A, T
H	A, C, T
B	G, C, T
V	A, C, G
D	A, G, T
N	A, C, G, T

Supplementary Table 3 - Macromolecular interfaces driven by trimerizer Helicons

**Buried Interface (Å^2)**	**Helicon:β-catenin**	**Helicon:MDM2**	**MDM2:β-catenin**
H330-mediated MDM2:β-catenin interaction	640	700	340
H332-mediated MDM2:β-catenin interaction	280	780	400

References

1. Li, K. *et al.* De novo mapping of alpha-helix recognition sites on protein surfaces using unbiased libraries. *Proc National Acad Sci* **119**, e2210435119 (2022).

2. Chen, Z. *et al.* A Tunable Brake for HECT Ubiquitin Ligases. *Mol. Cell* **66**, 345-357.e6 (2017).

3. Zhang, W. *et al.* System-Wide Modulation of HECT E3 Ligases with Selective Ubiquitin Variant Probes. *Mol. Cell* **62**, 121–136 (2016).

4. Han, X. *et al.* Discovery of ARD-69 as a Highly Potent Proteolysis Targeting Chimera (PROTAC) Degrader of Androgen Receptor (AR) for the Treatment of Prostate Cancer. *J Med Chem* **62**, 941–964 (2019).

5. Frost, J. *et al.* Potent and selective chemical probe of hypoxic signalling downstream of HIF-α hydroxylation via VHL inhibition. *Nat Commun* **7**, 13312 (2016).

6. Lumpkin, R. J., Baker, R. W., Leschziner, A. E. & Komives, E. A. Structure and dynamics of the ASB9 CUL-RING E3 Ligase. *Nat. Commun.* **11**, 2866 (2020).

7. Cardote, T. A. F., Gadd, M. S. & Ciulli, A. Crystal Structure of the Cul2-Rbx1-EloBC-VHL Ubiquitin Ligase Complex. *Structure* **25**, 901-911.e3 (2017).

8. Zheng, N. *et al.* Structure of the Cul1–Rbx1–Skp1–F boxSkp2 SCF ubiquitin ligase complex. *Nature* **416**, 703–709 (2002).

9. Angers, S. *et al.* Molecular architecture and assembly of the DDB1–CUL4A ubiquitin ligase machinery. *Nature* **443**, 590–593 (2006).

10. Chang, Y. S. *et al.* Stapled alpha-helical peptide drug development: a potent dual inhibitor of MDM2 and MDMX for p53-dependent cancer therapy. *Proc National Acad Sci* **110**, E3445-54 (2013).
