# Supplementary material for: Recognition and reprogramming of E3 ubiquitin ligase surfaces by α-helical peptides
Source: Nat Commun. 2023 Nov 1;14:6992. doi: 10.1038/s41467-023-42395-z (PMC10620186; doi:10.1038/s41467-023-42395-z)
Supplement: Supplementary file 3 — Reporting Summary [file 41467_2023_42395_MOESM3_ESM.pdf]

nature portfolio

Corresponding author(s): John McGee and Gregory Verdine

Last updated by author(s): Sep 22, 2023

Reporting Summary

Nature Portfolio wishes to improve the reproducibility of the work that we publish. This form provides structure for consistency and transparency in reporting. For further information on Nature Portfolio policies, see our Editorial Policies and the Editorial Policy Checklist.

Statistics

For all statistical analyses, confirm that the following items are present in the figure legend, table legend, main text, or Methods section.

n/a	Confirmed	
☐	☒	The exact sample size (*n*) for each experimental group/condition, given as a discrete number and unit of measurement
☐	☒	A statement on whether measurements were taken from distinct samples or whether the same sample was measured repeatedly
☒	☐	The statistical test(s) used AND whether they are one- or two-sided<br>*Only common tests should be described solely by name; describe more complex techniques in the Methods section.*
☒	☐	A description of all covariates tested
☒	☐	A description of any assumptions or corrections, such as tests of normality and adjustment for multiple comparisons
☐	☒	A full description of the statistical parameters including central tendency (e.g. means) or other basic estimates (e.g. regression coefficient) AND variation (e.g. standard deviation) or associated estimates of uncertainty (e.g. confidence intervals)
☒	☐	For null hypothesis testing, the test statistic (e.g. *F*, *t*, *r*) with confidence intervals, effect sizes, degrees of freedom and *P* value noted<br>*Give P values as exact values whenever suitable.*
☒	☐	For Bayesian analysis, information on the choice of priors and Markov chain Monte Carlo settings
☒	☐	For hierarchical and complex designs, identification of the appropriate level for tests and full reporting of outcomes
☒	☐	Estimates of effect sizes (e.g. Cohen's *d*, Pearson's *r*), indicating how they were calculated

Our web collection on statistics for biologists contains articles on many of the points above.

Software and code

Policy information about availability of computer code

Data collection	Illumina NovaSeq platform using a 2x150 bp high-output kit (Illumina) for next-generation sequencing<br>Biacore 8K (Cytiva) and Biacore S200 (Cytiva) instruments for SPR studies<br>GloMax Discover luminometer (Promega) for ELISA assays<br>Mosquito LV (SPT Labtech) for automation<br>MultiDrop Combi (Thermo) for automation<br>CLARIOstar and PheraStar plate readers (BMG Labtech) for fluorescence polarization and TR-FRET studies
Data analysis	Biacore Insight Evaluation Software v3.0 for Biacore 8K and Biacore S200<br>Discovery Wrokbench v4.0 for the GloMax Discover luminometer<br>Mosquito software v3.14.7 for the Mosquito LV.<br>MARS software v3.4.2 on the CLARIOstar and PheraStar plate readers.<br>Structural data processing and refinement used ccp4 7.1, Coot 0.8.9.2 and PHENIX 1.18. Structural model figures displayed using PyMOL 2.3.4.<br>GraphPad Prism 9.2 to generate data graphs

For manuscripts utilizing custom algorithms or software that are central to the research but not yet described in published literature, software must be made available to editors and reviewers. We strongly encourage code deposition in a community repository (e.g. GitHub). See the Nature Portfolio guidelines for submitting code & software for further information.

Data

Policy information about availability of data

All manuscripts must include a data availability statement. This statement should provide the following information, where applicable:

- Accession codes, unique identifiers, or web links for publicly available datasets
- A description of any restrictions on data availability
- For clinical datasets or third party data, please ensure that the statement adheres to our policy

Atomic coordinates and structure factors have been deposited in the Protein Data Bank with accession codes 8EI9 [https://www.rcsb.org/structure/8EI9], 8EIA [https://www.rcsb.org/structure/8EIA], 8EIB [https://www.rcsb.org/structure/8EIB], 8EIC [https://www.rcsb.org/structure/8EIC], 8EHZ [https://www.rcsb.org/structure/8EHZ], 8EI0 [https://www.rcsb.org/structure/8EI0], 8EI1 [https://www.rcsb.org/structure/8EI1], 8EI2 [https://www.rcsb.org/structure/8EI2], 8EI3 [https://www.rcsb.org/structure/8EI3], 8EI4 [https://www.rcsb.org/structure/8EI4], 8EI5 [https://www.rcsb.org/structure/8EI5], 8EI6 [https://www.rcsb.org/structure/8EI6], 8EI7 [https://www.rcsb.org/structure/8EI7], and 8EI8 [https://www.rcsb.org/structure/8EI8]. The DNA sequencing data acquired in the screening efforts have been deposited in the NCBI Sequence Read Archive under accession code PRJNA1019768 [https://www.ncbi.nlm.nih.gov/bioproject/PRJNA1019768]. All data needed to evaluate the conclusions of the study are present in the paper and the Supplementary files. Requests for materials should be addressed to jmcgee@fogpharma.com.

Research involving human participants, their data, or biological material

Policy information about studies with human participants or human data. See also policy information about sex, gender (identity/presentation), and sexual orientation and race, ethnicity and racism.

Reporting on sex and gender	n/a
Reporting on race, ethnicity, or other socially relevant groupings	n/a
Population characteristics	n/a
Recruitment	n/a
Ethics oversight	n/a

Note that full information on the approval of the study protocol must also be provided in the manuscript.

Field-specific reporting

Please select the one below that is the best fit for your research. If you are not sure, read the appropriate sections before making your selection.

☒ Life sciences ☐ Behavioural & social sciences ☐ Ecological, evolutionary & environmental sciences

For a reference copy of the document with all sections, see nature.com/documents/nr-reporting-summary-flat.pdf

Life sciences study design

All studies must disclose on these points even when the disclosure is negative.

Sample size	For biochemistry experiments, n=2 was the ideal minimal replicate number. This was determined to be sufficient based on the low observed variability between treated conditions and/or internal controls. n = 1 is used sparingly, but with the same rationale. All exact sample sizes are stated in the figure legends. For screening assays, targets were screened at least at five concentrations.
Data exclusions	No data were excluded, unless mentioned otherwise
Replication	All biochemistry experiments were conducted 2-3 times. Screening experiments had one replicate pair for each target. All attempts at replication in this study were successful.
Randomization	For the biochemistry and screening experiments, the samples were not randomly assigned, so there was no need for group allocation or randomization because all samples were consistently and independently measured in a controlled manner.
Blinding	For biochemistry experiments, blinding is not applicable because researchers need to verify samples and controls for each experiment. However, whenever feasible, an additional one or two researchers confirmed the results. Same statement applies to screening experiments.

Reporting for specific materials, systems and methods

We require information from authors about some types of materials, experimental systems and methods used in many studies. Here, indicate whether each material, system or method listed is relevant to your study. If you are not sure if a list item applies to your research, read the appropriate section before selecting a response.

Materials & experimental systems

n/a	Involved in the study
☒	☐ Antibodies
☒	☐ Eukaryotic cell lines
☒	☐ Palaeontology and archaeology
☒	☐ Animals and other organisms
☒	☐ Clinical data
☒	☐ Dual use research of concern
☒	☐ Plants

Methods

n/a	Involved in the study
☒	☐ ChIP-seq
☒	☐ Flow cytometry
☒	☐ MRI-based neuroimaging
