# Supplementary material for: Recognition and reprogramming of E3 ubiquitin ligase surfaces by α-helical peptides
Source: Nat Commun. 2023 Nov 1;14:6992. doi: 10.1038/s41467-023-42395-z (PMC10620186; doi:10.1038/s41467-023-42395-z)
Supplement: Supplementary file 4 — Description of Additional Supplementary Files [file 41467_2023_42395_MOESM4_ESM.pdf]

Description of Additional Supplementary Files

File Name: Supplementary Data 1
Description: Phage counts for each target define hits and clusters (C71- C94) from phage screening across all individual E3s and E3-target complexes.

File Name: Supplementary Data 2
Description: Structural data collection and refinement statistics for all reported structures.
